# The Impact of Plant Essential Oils on the Growth of the Pathogens *Botrytis cinerea*, *Fusarium solani*, and *Phytophthora pseudocryptogea*

**DOI:** 10.3390/life14070817

**Published:** 2024-06-27

**Authors:** Petya K. Christova, Ana M. Dobreva, Anatoli G. Dzhurmanski, Ivayla N. Dincheva, Stela D. Dimkova, Nadejda G. Zapryanova

**Affiliations:** 1 Agrobioinstitute, Agricultural Academy, 8 “Dragan Tsankov” Blvd., 1164 Sofia, Bulgaria; ivadincheva@yahoo.com; 2 Institute of Aromatic Plants, Agricultural Academy, 49 Osvobozhdenie Blvd., 6100 Kazanlak, Bulgaria; 3 Institute of Ornamental and Medicinal Plants, Agricultural Academy, Negovan, 1222 Sofia, Bulgaria

**Keywords:** *Monarda*, *Mentha*, *Tagetes*, EOs, inhibition, antifungal

## Abstract

Essential oils (EOs) extracted from aromatic and medicinal plants have the potential to inhibit the growth of various pathogens and, thus, be useful in the control of dangerous diseases. The application of environmentally friendly approaches to protect agricultural and forestry ecosystems from invasive and hazardous species has become more significant in last decades. Therefore, the identification and characterization of essential oils with a strong inhibitory activity against aggressive and widespread pathogens are of key importance in plant protection research. The main purpose of our study is to evaluate the impact of essential oils originating from different genotypes of bee balm, mint, and marigold on *Botrytis cinerea*, *Fusarium solani*, and *Phytophthora pseudocryptogea*. Twelve essential oils, including five EOs originating from *Monarda fistulosa*, one oil each from *Monarda russeliana*, *Mentha longifolia*, *Mentha piperita*, *Tagetes patula*, and *Tagetes erecta*, and two EOs from *Tagetes tenuifolia* were derived by steam or water distillation. The chemical composition of the tested EOs was determined by GS-MS analyses and their corresponding chemotypes were identified. The most effective against all three pathogens were determined to be the EOs originating from *M. fistulosa* and *M. russeliana*. *B. cinerea*, and *P. pseudocryptogea* were also significantly affected by the *M. piperita* essential oil. The most efficient EOs involved in this investigation and their potential to control plant pathogens are discussed.

## 1. Introduction

*Botrytis cinerea* is a cosmopolitan fungus that infects more than 200 plant species and is one of the most destructive fungal pathogens globally. It is estimated that this fungus causes annual losses of up to USD 100 billion worldwide [1]. *Fusarium solani* is among the most common and economically important *Fusarium* species associated with soil-borne diseases in many agricultural crops. It has been reported as the most damaging root rot pathogen [2]. Although many *Phytophthora* species are known as saprophytes, hemibiotrophs, or opportunistic necrotrophs, some members of the genus are very dangerous invasive plant pathogens that cause devastating diseases on forest and agricultural ecosystems [3]. Controlling these pathogens is a challenge and requires continuous scientific research and the application of innovative methods. The use of essential oils for plant disease management is a preferred environmentally friendly approach.

A number of essential oils derived from aromatic and medicinal plant species have been proven to be effective against *B. cinerea*. Large-scale investigations of the antifungal activity of 38 EOs against 10 plant pathogens revealed that the most effective against *B. cinerea* are oils from palmarosa, oregano, clove, cinnamon, citronella, and thyme [4]. The efficiency of lemongrass (*Cymbopogon citrates*) and thyme (*Thymus capitatus*) oils for the inhibition of *B. cinerea* mycelial growth, as well as the germination of spores, have been proved too [5]. EOs from oregano (*Origanum vulgare*), thyme (*Thymus vulgaris*), and lemon (*Citrus limon*) have also demonstrated in vitro and in vivo activities against gray mold [6].

Several experiments evaluated the *in vitro* antifungal activity of different essential oils against *F. solani*. Effectiveness against the fungus showed EOs from jasmine (*Cestrum nocturnum*) and common wormwood (*Artemisia absinthium*) [7,8]. The EOs originating from lemongrass, citral, nerol, and thyme also demonstrated inhibitory activity against *F. solani* and the effect have been improved by increasing the applied concentrations [9]. EOs from citronella grass (*Cymbopogon nardus*), lemon grass (*Cymbopogon citrates*), and garlic (*Allium sativum*) were more efficient for controlling *F. solani* in comparison to oils from sweet marjoram (*Origanum majorana*), rosemary (*Rosmarinus officinalis*), black pepper (*Piper nigrum*), and copaiba (*Copaifera reticulate*) after evaluating their impact on mycelial growth, germination, and the sporulation of the fungus [10].

Studies on the possibility to manage pathogens from the genus *Phytophthora* through the application of EOs have been in progress in recent years. The effectiveness of different EOs against a number of *Phytophthora* species, including *P. capsici*, *P. infestance*, *P. cryptogea*, *P. nicotianae*, *P. cinnamomi,* and *P. parasitica* have been reported [9,11,12,13,14,15,16]. Some of these studies investigated the inhibitory effect of a wide variety of essential oils. An extensive analysis of fourteen commercial products against *P. capsici* resulted in the determination of EOs from oregano, palmarosa, red thyme, clove bud, cinnamon leaf, and citronella java as being the most effective inhibitors of the mycelial growth, followed by peppermint and spearmint, however, EOs of bay, rosemary, Spanish sage, sweet fennel, tea tree, and lavender had no effect at the tested concentrations [11]. One of the most devastating oomycete pathogens, *P. infestance*, was the object of a large-scale investigation that confirmed the potential of clove and thyme EOs to inhibit mycelial growth at the lowest concentration, followed by cinnamon, rosemary, and tea tree EOs [12]. In contrast, less effective against the invasive species were rosemary and tea tree EOs, while pepper EO was not active even at a higher concentration.

A number of plant species are used as sources of essential oils and bioactive compounds. The labiate genus *Monarda* L. (Lamiaceae) is native to North America and includes valuable oil-bearing and medicinal plants [17,18]. Another member of the Lamiaceae family, the genus *Mentha*, is distributed and cultivated worldwide, and includes aromatic herbs that are rich in essential oils [19]. The genus *Tagetes* belongs to the Asteraceae family and is native to America, but is widespread because of its ornamental value [20].

Essential oils are complex mixtures of chemically diverse compounds, and they share common properties: volatility and insolubility in water. The distillation method uses exactly these attributes. In turn, the distillation can use water (the plant material boils in an aqueous medium) or steam (the steam passes through the raw material). The first mode fully extracts the essential oil, but it is prolonged and less economical effective. It is used for matrixes with a minimal amount of essential oil or to obtain very expensive oils. The second mode is preferred at the industrial scale, because of its simplicity and effectiveness, although the yield is not perfect. Although there is no significant change in composition, the method of distillation still has an effect on the quantitative variation of individual components [21].

The purpose of our study was to examine the composition of 12 essential oils extracted from different genotypes of bee balm, mint, and marigold, and to evaluate their effect on the growth of three important plant pathogens: *B. cinerea*, *F. solani*, and *P. pseudocryptogea*. To the best of our knowledge, some of the most effective EOs involved in this investigation that have the potential to control the tested pathogens have not been reported until now.

## 2. Materials and Methods

### 2.1. Plant Material

The plants of bee balm (*Monarda* spp.), mint (*Mentha* spp.), and marigold (*Tagetes* spp.) that were used in this experimental work are listed and described in Table 1.

All the plants *M. fistulosa* (1–4), except for number 5 and *M. russeliana*, were grown in field conditions at the Experimental Field of the Institute for Roses and Aromatic Plants (IRAP), Kazanlak, Bulgaria (42.61°94′408″ N/25.39°29′576″ E, altitude of 407 m). The soils in the area were leached cinnamon forest, developed on old diluvial deposits, structureless with a good aeration and water permeability, with an acidic pH of 4.9, and poorly stocked with nitrogen 20.5 mg/1000 g, phosphorus 4.25 mg/100 g, potassium 21.75 mg/100 g, and a humus content of 1.8%. The planting pattern was 25 cm × 70 cm (within the row × between the rows). The areas were cultivated twice during the growing season. Fertilization was carried out in early spring with ammonium nitrate 100 kg/ha. In case of drought (below 65% maximum field moisture content), watering was carried out using a stationary drip system.

The plants of mint, marigold, and M. fistulosa number 5 were grown in the medicinal plant collection of the Institute of Ornamental and Medicinal Plants (IOMB) Negovan, Sofia, Bulgaria (42°44′42.78″ N/23°24′ 05.49″ E, altitude 524). The climate in the area is moderately continental with an average maximum monthly temperature of 27.5 °C. The experimental field is located near the river Iskar. The soil is alluvial-meadow with the following characteristics: pH—6.5 in KSI, humus content—1.9%, N_2_—from 1.6 to 2.1 mg/100 g of soil, K_2_O—from 5.0 to 7.0 mg/100 g soil, and P_2_O_5_—12 mg/100 g soil. In the collection, the plants were planted according to the scheme of 40 cm/70 cm (inside the row/between the rows). Fertilization and plant protection measures were not applied. The areas were cultivated three times during the growing season. Watering was regular, through an established drip system. Plant harvest was performed in the full flowering period (June–July).

### 2.2. Extraction of EOs

The fresh raw material was processed by distillation in a laboratory glass apparatus [22,23]. The process conditions were selected according to the kind of the material and its oil content. A steam distillation type was applied for samples from numbers 1 to 8, and number 10, as well as a water distillation for samples 9, 11, and 12 was used (Table 2). The model was chosen according the content of the essential oil in the raw material. The parameters of the process were: debit flow of 5–7 mL min^−1^, duration of 2 h, and distillate temperature of 28–30 °C. The quantity of the oil was measured in milliliters on the receiver scale. The oil obtained was dried over anhydrous Na_2_SO_4_, filtered, and stored in tightly closed dark vials at 4 °C until analysis. All the quantitative experiments were performed in three replicates.

### 2.3. GC–MS Analysis

The chemical composition of the EOs was analyzed by the gas chromatography technique coupled with mass spectrometry (GS-MS). The GC analysis was conducted on an Agilent 7890A system with an FID and a DB-5 capillary column (30 m × 0.25 mm, 0.25 μm). The temperature program ranged from 40 °C to 300 °C at a rate of 5 °C/min. Nitrogen was used as the carrier gas at a flow rate of 0.8 mL/min. The injector port and detector temperatures were set at 230 °C and 280 °C, respectively, and samples were injected with a split ratio of 30:1. The GC/MS analysis was carried out on an Agilent 7890A/5975C system, also with a DB-5 capillary column (30 m × 0.25 mm, 0.25 μm). The operating conditions matched those described for the GC analysis. Helium was the carrier gas, flowing at 0.8 mL/min. Mass spectra were acquired in electron impact (EI) mode at 70 eV, with a scan mass range of 40–400 m/z. The ionization source, transfer line, and injector temperatures were 230 °C, 280 °C, and 250 °C, respectively. Quantitative data were obtained by electronically integrating the FID peak areas. The components of the oil samples were identified based on their retention time, retention indices (relative to C_8_-C_40_ n-alkanes), and matching with the Adams and NIST’08 libraries, as well as compared with the existing literature data. The percentage composition of the identified compounds was calculated from the GC peak areas without using correction factors.

### 2.4. In Vitro Inhibition Test

Three plant pathogens (*B. cinerea*, *F. solani*, and *P. pseudocryptogea*) were cultivated in vitro and their response to 12 EOs originating from different subspecies of bee balm, mint, and marigold was evaluated by applying the agar diffusion method. Six essential oils from bee balm, five of them from *M. fistulosa* (M.f. 1, 2, 3, 4, and 5) and one from *M. russeliana* (M.r.), two mint EOs, *M. piperita* (M.p.) and *M. longifolia* (M.l.), and four oils from marigold, including two samples eluted from *T. tenuifolia* (T.t.1, and 2) and one each oil of *T. erecta* (T.e.) and *T. patula* (T.p.), were used in the experiment. The bee balm oils were applied in three doses (1, 2, and 3 µL), whereas the rest of the EOs were tested in four doses (2, 3, 4, and 5 µL) according to the results from preliminary experiments on their efficiency and the amount of oils available. All isolates that were tested are part of the fungal collection of AgroBioInstitute, Sofia, Bulgaria.

Mycelia plugs (5 mm) from 7-day-old cultures of each pathogen were transferred to fresh potato dextrose agar (PDA) in Petri dishes (90 mm diameter). The tested EOs in corresponding doses were applied approximately 2 cm from the mycelium plug. The same cultures of the pathogens incubated on PDA without essential oils were used as controls. All cultures were grown at 20 °C in the dark and the results were counted after 5 days for *B. cinerea*, and after 10 days for *F. solani* and *P. pseudocryptogea*. The effect of the EOs on mycelium growth was evaluated by measuring the size of the developed colonies (mm) in the two orthogonal diameters.

### 2.5. Statistical Analysis

Each variant of the treatments in the *in vitro* tests was prepared in 3 replicates and the experiment was performed 2 times. The statistical significance of the differences between the values in the conducted pathogenicity tests was assessed by *t*-test one way ANOVA at a probability level of *p* ≤ 0.05.

## 3. Results

### 3.1. Extraction and Chemical Composition of EOs

The results of the EOs’ extraction from the tested plants are presented in Table 2. The yield for different samples was performed as essential oil content of the raw material. The EO content of the tested *Monarda* spp. plants varied from 0.45 to 0.75%, and was even lower for *M. russeliana* (0.36%). The yield differences for the two mint species was almost double: 0.26% for *M. longifolia* and 0.55% for *M. piperita*. The lowest EO content was extracted from the marigold plants, starting from 0.03% and 0.04% for *T. erecta* and *T. patula*, respectively, and reaching up to 0.14–0.22% for the both *T. tenuifolia* samples. In this genotype, the oil is in minimal quantities, and this predetermined the use of water distillation. In *T. erecta* and *T. patula*, the content is in a microquantity, but in *T. tenuifolia*, it is in relatively higher levels, and this allowed for applying the two extraction models—water and steam distillation. The result confirmed that water distillation completely extracted the oil and the yield was 50% higher.

The chemical compositions of the extracted EOs from the tested bee balm, mint, and marigold plants are presented in Table 3, Table 4 and Table 5, respectively.

A total number of 36 compounds were identified in the analyzed samples of the bee balm plants (Table 3). Thymol was the main component in *M. fistulosa* numbers 1 and 2, as well as in *M. russeliana*, represented by 52%, 57%, and 65%, respectively. In contrast, for the other three samples from *M. fistulosa* (3, 4, and 5), a predominant amount of carvacrol was counted (78%, 55%, and 71%, respectively). In addition, only in samples M.f. 3, 4, and 5, carvacrol methyl ether was also in an increased amount (5–7%). Other representative compounds with a higher content in the tested bee balm samples were p-cymene (7–20%) and γ-terpinene (3–18%).

Altogether, 71 chemical compounds were identified in the EOs from the both mint plants (Table 4). They were characterized by significant differences in their chemical composition. *M. longifolia* is distinguished by an increased content of (Z)-sabinene hydrate (17%), menthone (15%), γ-terpinene (8%), 1-terpinen-4-ol (8%), and germacrene D (7%). Largest groups of constituents in *M. piperita* were germacrene D (22%), β-caryophyllene (20%), and piperitone (8%).

A total number of 29 compounds were identified in the analyzed samples from the tested marigold plants (Table 5). The largest group of constituents in *T*. *tenuifolia* 1 and 2 was trans-ocimenone, represented by 41% and 49% of TIC, respectively. Other compounds in a higher amount in the both samples were cis-ocimenone (12% and 7%, respectively), limonene (10% and 4%, respectively), and dihydrotaghetone (8% and 9%, respectively). Some of these chemical compounds were also the main elements in the EOs extracted from *T. patula*, including cis-ocimenone (13%) and trans-ocimenone (9%), as well as some others like β-trans-ocimene (16%), terpinolene (13%), and piperitenone (11%). The largest group of constituents in *T. erecta* was terpinolene (28%), followed by piperitone (18%), limonene (9%), and dihydrotaghetone (8%). It can be concluded that there was a certain difference in composition of T.e.1 and T.e.2 regarding the oil obtained by water and steam distillation: limonene was 50% more in the T.e.1 sample, but specific trans-tagetone and cis-tagetone were significantly more in T.e.2. The main component trans-ocimenone showed no significant difference.

### 3.2. Impact of EOs on Plant Pathogens

**Figure 1:**
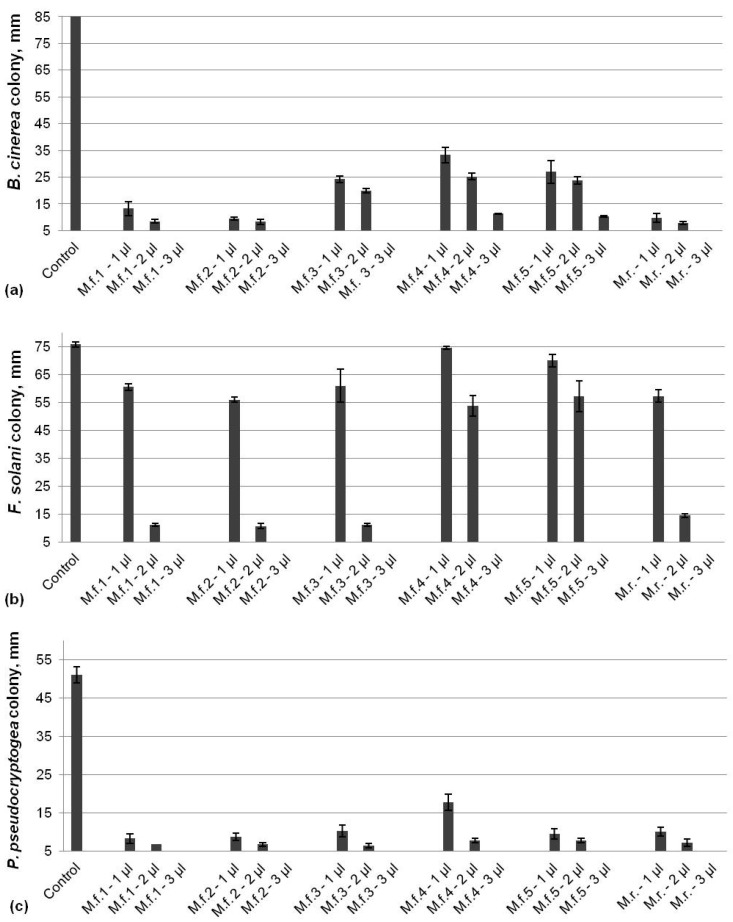
Inhibitory activity of EOs from bee balm plants against *B. cinerea* (**a**), *F. solani* (**b**), and *P. pseudocryptogea* (**c**).

Evaluation of the antifungal activity of six EOs extracted from bee balm plants against *B. cinerea*, *F. solani*, and *P. pseudocryptogea* showed significant inhibition of the tested pathogens (Figure 1). All EOs led to a more than 60% reduction in *B. cinerea* colony size compared to the control in a dose-dependent manner (Figure 1a). The fungal growth was inhibited by about 90% by the lowest dose (1 µL) of three EOs (M.f. 1, M.f. 2, and M.r.) that were determined as the most effective against *B. cinerea*. The highest applied dose (3 µL) of all EOs resulted in the total inhibition of the pathogen with the exception of the oils M.f. 4 and M.f. 5, which resulted in a 90% reduction in the colony size. In contrast to *B. cinerea*, *F. solani* was less sensitive to the application of lower doses of bee balm oils and the fungal growth was reduced from 20 to 30% by the EOs M.f. 1, 2, 3, and M.r., with almost no effect by M.f. 4 and M.f. 5 (Figure 1b). However, the application of 2 µL of EOs led to a significant reduction in the colony size (about 90%) with an exception again of M.f. 4 and M.f. 5, which were less effective. A total inhibition of *F. solani* was achieved by using 3 µL of all tested EOs. All bee balm EOs demonstrated a high potential to inhibit the growth of oomycetes species *P. pseudocryptogea* at low doses (Figure 1c). The size of colonies was reduced about 85% by 1 µL of essential oils, with the exception of M.f. 4 (65%), and a total inhibition by all EOs after the application of 3 µL was observed.

**Figure 2:**
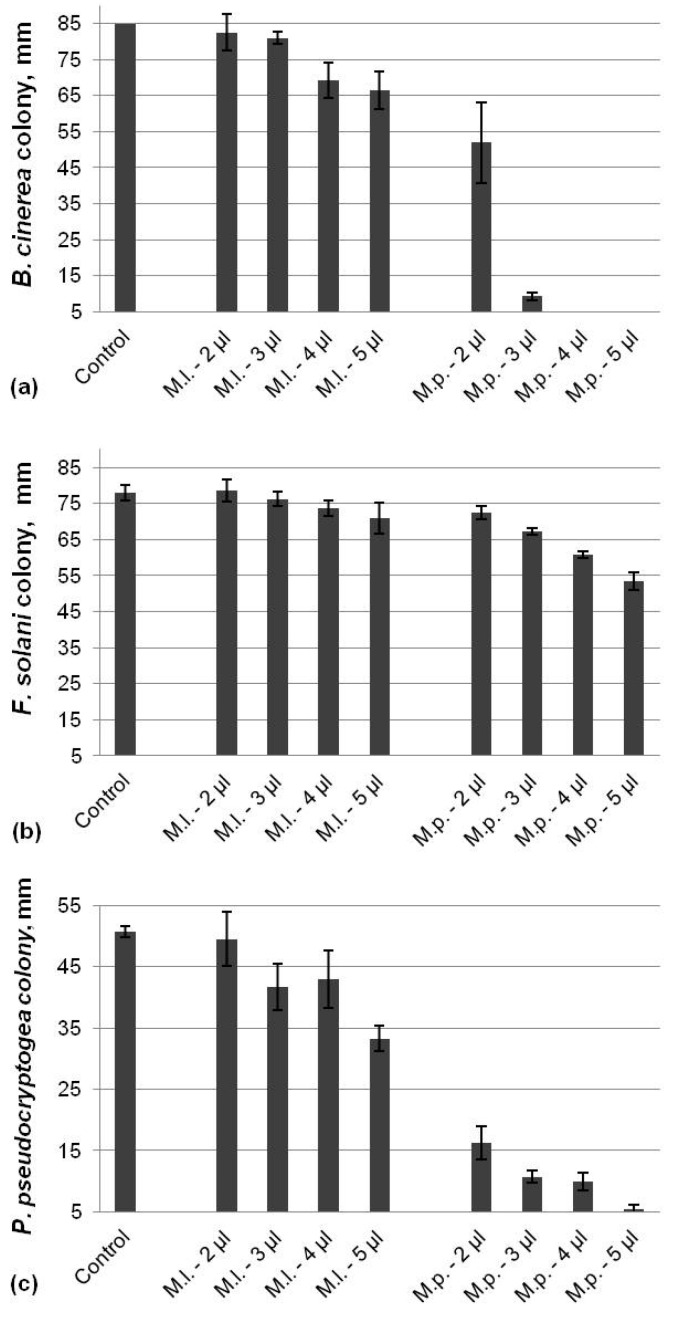
Effect of EOs from mint plants against *B. cinerea* (**a**), *F. solani* (**b**), and *P. pseudocryptogea* (**c**).

Both EOs eluted from a mint species demonstrated totally different antifungal activity against *B. cinerea*, *F. solani*, and *P. pseudocryptogea* (Figure 2). The EO from *M. piperita* was able to significantly inhibit the growth of *B. cinerea* at concentrations of more than 3 µL, whereas the oil from *M. longifolia* was less effective and led to a barely 21% reduction in the mycelial colony at the highest applied dose (Figure 2a). The slightest antifungal activity of the mint oils was monitored for *F. solani* (Figure 2b). The application of 5 µL of EOs resulted in a fungal growth inhibition of 31% by the *M. piperita* oil and only 9% for the EO from *M. longifolia*. Similarly to *B. cinerea*, the two mint oils demonstrated notably different potential to inhibit *P. pseudocryptogea* (Figure 2c). A significant reduction in the mycelia growth was achieved by the *M. piperita* EO, reaching 88% at the maximum applied dose compared to the control. The same concentration of the oil from *M. longifolia* led to a barely 34% inhibition of the colony size.

**Figure 3:**
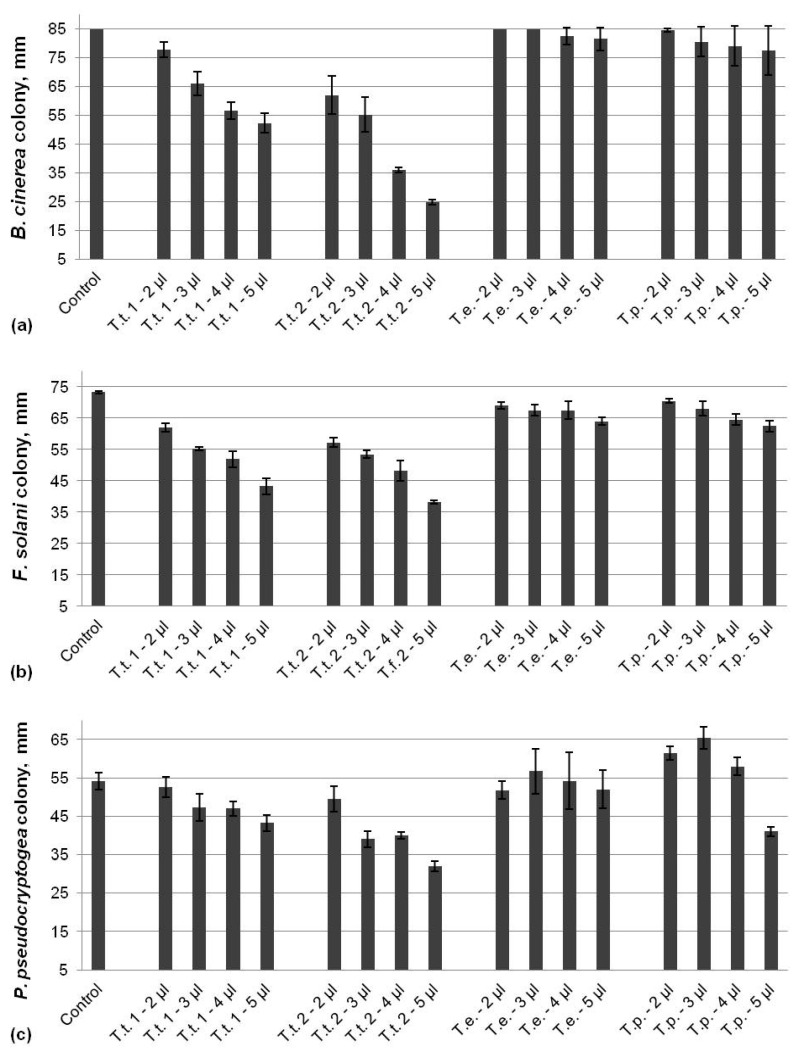
Activity of EOs from marigold against *B. cinerea* (**a**), *F. solani* (**b**), and *P. pseudocryptogea* (**c**).

Two of the tested marigold oils originating from *T. tenuifolia* demonstrated dose-dependent antifungal activities against all three analyzed pathogens, while the effect of the other two oils extracted from *T. erecta* and *T. patula* was not so eloquent (Figure 3). The inhibition of *B. cinerea* varied from 8% to 39% for the T.t. 1 EO and from 27% to 71% for the T.t. 2 EO, and the effect increased with increasing the applied dose. No significant impact on the pathogen growth by the *T. erecta* and *T. patula* oils at any of the tested concentrations was monitored (Figure 3a). There was a reduction in *F. solani* colonies reaching up to 41% for T.t. 1 and 48% for T.t. 2 EOs at the highest applied dose (Figure 3b). The inhibition of the fungal growth by the both EOs from *T. erecta* and *T. patula* was negligible, with a maximum of only 14% compared to the control. The inhibition of *P. pseudocryptogea* was up to 20% by T.t. 1 and 40% for T.t. 2 EOs, which shows the potential for more efficiency at higher doses (Figure 3c). No inhibitory effect on the oomycetes growth by the essential oil from *T. erecta* at the tested concentrations was observed. However, low doses of the *T. patula* EO led to the induction of the mycelia growth to 115%, 122%, and 107% after the application of 2, 3, and 4 µL, respectively, whereas 5 µL EOs resulted in reduction in the colony size with 24% compared to the control.

## 4. Discussion

In this study, 12 essential oils derived from three different plant species, bee balm, mint, and marigold, are presented. All the values for the extracted EOs from the tested plants showed that the yield depended on the genotype. *Monarda* spp. were the most abundant, with levels from 0.36% to 0.75% (Table 2). Between the *M. fistulosa* samples, it was obvious that their origin was of the greatest of importance for the yield. The samples originating in USA, Slovenia, and Negovan demonstrated a high potential for oil accumulation, but they could not reach the selected samples of the varieties. The cultivar “Mona” revealed the levels that have been declared [24]. The slightly higher content in the overblown sample M.f. 4 was related to the lower moisture in the plant material. The Ukraine varieties “Futuna” and “Premiera” of *M. fistulosa* referred the same range of essential oil content: 0.7–0.8% [25]. There were other data about higher and lower yields of this species, but they concerned dry material or different geographical origin [17,18,26]. The subspecies *M. russeliana* had the lowest essential oil content. It is not of economic interest and the literature lacks data on its extract.

Concerning mint, the yield difference was almost double, and this is a reasonable result given the genotypes [27].

*Tagetes* spp. showed significantly lower levels of oil yield varying from 0.03% to 0.22% (Table 5). The same oil content from *T. patula* and *T. erecta* was reported in other study [28]. Although grown as ornamentals plants, the *T. tenuifolia* samples showed a relatively higher yield. T.t. 1 was isolated after water distillation, which fully exhausted the material and was why the content was 52% higher than T.t. 2. The steam distillation gave less yield, but the essential oil was closer to the industrial conditions and practices, respectively [23].

According to the results from the conducted GC-MS analysis, the tested plants *M. fistulosa* 1 and 2 and *M. russeliana* are thymol chemotypes, whereas *M. fistulosa* 3, 4, and 5 are carvacrol chemotype (Table 3). Both chemotypes of *Monarda* spp. have been reported previously by other authors and our former study [17,18,29]. An investigation of the main compounds of the carvacrol chemotype *M. fistulosa* in two different years by Grzeszczuk et al. [17] showed more than a two times lower content of carvacrol (28% for 2015 and 24% for 2016) compared to the tested in our study samples (51–78%). Similarly, for the thymol chemotype *M. fistulosa*, contents of 28–33% thymol have been reported [18], whereas the same main compound in *M. fistulosa* 1 and 2 was 52% and 67%, respectively. Despite belonging to different chemotypes, these oils demonstrated a significant inhibitory effect against the tested pathogens. Although the carvacrol EOs (M.f. 3, 4, and 5) had the lowest antimicrobial activity against *B. cinerea* and partially *F. solani* (except for M.f.3), this was not the case for the activity against *P. pseudocryptogea*.

Both mint species demonstrated a complete difference in the chemical composition of their EOs, as the main components in *M. longifolia* were (Z)-sabinene hydrate (17%) and menthone (15%), whereas in *M. piperita*, germacrene D (22%) and β-caryophyllene (20%) prevailed. It can be assumed that these significant differences affect their properties, including their inhibitory activity against the tested pathogens, which was completely dissimilar (Figure 2). This great variety in the chemical compositions of EOs derived from different types of mint is expected, as it has been found by other authors as well [30,31].

Among the main components in the both EOs of *T. tenuifolia* were trans-ocimenone and cis-ocimenone, which stood out by over 40% of TIC (Table 5). A high content of (E)-ocimenone has been previously reported for EOs from two other *T. tenuifolia* plants, respectively, at 34% and 47% [32]. The main compound in the essential oil of *T. erecta* was terpinolene (28%), followed by piperitone (18%), which is similar to data published before for the same marigold species in which α-terpinolene was the major compound (18%), but with a lower percentage content, followed by (E)-ocymenone (13%) [33]. The chemical composition of the essential oil from *T. patula* combined the main components of the two other analyzed marigold species, including cis-ocimenone (13.26%) and trans-ocimenone (9.3%), like EOs from *T. tenuifolia*, but high contents of terpinolene (13.4%) and piperitenone (11.05%) similar to the dominant ingredients into the *T. erecta* oil were also identified. The highest percentage in the EO of *T. patula* belonged to β-trans-ocimene (16.4%), a component that is sparsely represented in the other three species (1.9–4.4%). According to all the experiments conducted, the EOs from the three *Tagetes* spp. differed not only in composition, but also in their activity against the studied pathogens. In addition to the inhibitory and neutral effect of the tested EOs, the growth stimulation of *P. pseudocryptogea* by the essential oil of *T. patula* was observed. However, it is currently not possible to determine which component is responsible for this property. One possibility for the induction of a mycelium growth is the existence of a constituent that is in high concentration only in *T. patula* compared to the EOs derived from other marigold species, such as β-trans-ocimene. On the other hand, the possibility that this effect is due to some of the components that are highly active even at low concentrations is not excluded, so future studies are needed to verify these suggestions.

The most effective against the tested plant pathogens EOs involved in this study originated from *M. fistulosa* and *M. russeliana* (Figure 1). They had the potential for the total inhibition of all three pathogens (*B. cinerea*, *F. solani*, and *P. pseudocryptogea*), and the majority of the tested extracts led to complete suppression at a low dose. Our previous investigation proved a strong antifungal activity against different plant pathogens of essential oil extracted from *M. didyma* [29]. An evaluation the effect of essential oils from *M. didyma*, *M. didyma* var. 80-1A, and *M. fistulosa* against *B. cinerea* by other authors also showed variable and dose-dependent inhibitory activities [34]. *M. didyma* demonstrated a significantly higher inhibitory effect and the lowest activity was achieved by *M. fistulosa*, which managed to inhibit the mycelial growth completely, only at the highest tested concentration [34]. A fungicidal effect of EOs from *M. didyma* on *B. cinerea* and for EOs from *M. fistulosa* against *Colletotrichum musae* and *Lasiodiplodia theobromae* have also been reported [35,36]. To the best of our knowledge, the ability of EOs from *M. fistulosa* and *M. russeliana* to inhibit the growth of *F. solani* and *P. pseudocryptogea* is an innovative option to control these plant pathogens.

Of the two analyzed mint species, the EOs from *M. piperita* showed a significant ability to inhibit the growth of *B. cinerea* and *P. pseudocryptogea* (Figure 2). In contrast, recently published data for the evaluation of twelve different essential oils against eight fungal pathogens revealed that the *M. piperita* EO fails to suppress the growth of *B. cinerea* [37]. Our experiments showed that the other tested fungal pathogen, *F. solani*, was much less affected by the *M. piperita* EO, which is in agreement with newly published data for similar applied doses [38,39]. It is noteworthy that the mint oils were effective against the oomycete species *P. pseudocryptogea*, since there ae limited data concerning the inhibitory activity of EOs from *Mentha* spp. against members of the genus *Phytophthora*. The ability of *M. piperita* and *P. spicata* to suppress the mycelial growth of *P. capsici* with a moderate effectiveness in EO amended agar media experiments has been demonstrated, whereas in volatility tests, peppermint EOs were less efficient and no effect of spearmint oils was observed [11]. Our results showed that *P. pseudocryptogea* is notably more sensitive to the EOs from *M. longifolia* compared to both tested fungi at the applied doses. These data indicate the importance of evaluating any combination between plant pathogens and essential oils with diverse origins.

Both essential oils from *T. tenuifolia* demonstrated dose-dependent antifungal activity against all three pathogens (Figure 3). However, the tested EOs from *T. erecta* and *T. patula* were not able to inhibit *B. cinerea,* and no significant effect on *F. solani* was also observed. In contrast, the potential of the EO from *T. patula* to suppress *B. cinerea* has been reported earlier [40,41]. A dose-dependent manner was observed, however, the applied doses in these studies were much higher than those used in our investigation. Therefore, we can assume that a larger amount of the tested marigold oils can lead to a more significant effect on *B. cinerea*. On the other hand, achieving a high efficiency with a minimum dose of the oil is preferred for practical purposes. Similar to our data for *F. solani*, a limited effect on the fungal growth by oils from other marigold species, *T. minuta*, has been published previously [42,43]. Similar to the inhibitory activity of the both EOs from *T. tenuifolia* to the tested fungal pathogens, their potential to control the oomycete species *P. pseudocryptogea* was also documented. Surprisingly, an induction of the pathogen growth at low doses was caused by the oil originating from *T. patula*. The effect of EOs from *T. patula* and *T. erecta* against six bacterial and three fungal species has been evaluated recently [44]. In these experiments, inhibitory activity against another oomycete, *Phytophthora erythroseptica*, was shown only by flower extracts from *T. patula*, however, no data for stimulating effect of the tested marigold oils on the *Phytophthora* species have been reported.

A number of studies have pointed out the relationship of specific components and inhibitory activity against important plant pathogens. Recently, published data demonstrated a significant antifungal effect of thymol and carvacrol against *B. cinerea* by disruption and distortion the mycelia of the fungus [45]. It could be suggested that the ability to totally inhibit the growth of the same fungal pathogen by *Monarda* EOs from both chemotypes (thymol and carvacrol) presented in our study was conditioned by a similar mechanism of action. Moreover, the potential of thymol and carvacrol to completely suppress gray mold decay has been also proven in vivo [46], indicating their potential as disease control agents.

An effective suppression of the postharvest grey mold disease on tomato has been achieved by *Cupressus sempervirens* essential oils and their major compounds [47]. In this study, germacrene D has been reported as the most representative compound. In addition, the mixture of α-pipene and β-caryophyllene has been more active against *B. cinerea* than synthetic fungicide, which confirms the potential of these compounds in the control of the pathogen. Similarly, our experimental data showed that *M. piperita* EOs that are rich in germacrene D and β-caryophyllene are much more effective against *B. cinerea* of the two tested mint extracts. Summarizing the data from our results and previous publications demonstrates the ability of plant EOs to be used for the control of important pathogens.

## Figures and Tables

**Table 1:** Plant material.

No.	Plant Name	Origin	Place and Year of Cultivation	Vegetative Stage at Harvesting
1	*Monarda fistulosa* L.; M.f.1	USA; Missouri botanical garden, reg. No. 125/2005.	IRAP, Kazanlak; 2021	Flowering end
2	*Monarda fistulosa* L.; M.f.2	Slovenia, Botanici Vat. Univerze, reg. No. 234/218.	IRAP, Kazanlak; 2021	Flowering end
3	*Monarda fistulosa L.*, cultivar Mona; M.f.3	Bulgaria; Selected through individual selection of seed progeny. The variety is maintained by clonal selection.	IRAP, Kazanlak; 2021	Flowering end
4	*Monarda fistulosa* L., cultivar Mona; M.f.4	Bulgaria; Selected through individual selection of seed progeny. The variety is maintained by clonal selection.	IRAP, Kazanlak; 2021	Over blown
5	*Monarda fistulosa* L.; M.f.5	United Kingdom, reg. No. 5/2020.	IOMP, Negovan; 2021	Flowering
6	*Monarda russeliana* (Russel’s Horsemint); M.r.	Germany; Botanischer garden de TU Braunschweig, reg. No. 1506/2018	IRAP, Kazanlak; 2021	Flowering end
7	*Mentha longifolia*; M.l.	Bulgaria; Local species from the area of Negovan.	IOMP, Negovan; 2021	Flowering
8	*Mentha piperita*; M.p.	Bulgaria; Local species from the area of Dryanovo, Gabrovo province, reg. No. 1/2019.	IOMP, Negovan; 2021	Flowering
9	*Tagetes tenuifolia*; T.t.1	Bulgaria; An old local variety from the area of Negovan.	IOMP, Negovan; 2022	Flowering
10	*Tagetes tenuifolia*; T.t.2	Bulgaria; An old local variety from the area of Negovan.	IOMP, Negovan; 2022	Flowering
11	*Tagetes erecta*; T.e.	Bulgaria; An old local variety from the area of willage Dolen, Blagoegrad province, reg. No. 10/2021.	IOMP, Negovan; 2022	Flowering
12	*Tagetes patula*, cultivar Usmivka; T.p.	Bulgaria; Selected through individual selection of seed progeny.	IOMP, Negovan; 2021	Flowering

**Table 2:** Essential oil distillation type and yield.

No.	Sample	Distillation Type	EO Content, %
1	*M. fistulosa*; M.f.1	Steam	0.491 ± 0.022
2	*M. fistulosa*; M.f.2	Steam	0.670 ± 0.000
3	*M. fistulosa cv. Mona*; M.f.3	Steam	0.700 ± 0.055
4	*M. fistulosa cv. Mona*; M.f.4	Steam	0.746 ± 0.052
5	*M. fistulosa*; M.f.5	Steam	0.450 ± 0.077
6	*M. russeliana*; M.r.	Steam	0.359 ± 0.019
7	*M. longifolia*; M.l.	Steam	0.262 ± 0.022
8	*M. piperita*; M.p.	Steam	0.545 ± 0.012
9	*T. tenuifolia*; T.t.1	Water	0.217 ± 0.005
10	*T. tenuifolia*; T.t.2	Steam	0.143 ± 0.004
11	*T. erecta*; T.e.	Water	0.033 ± 0.003
12	*T. patula*; T.p.	Water	0.040 ± 0.009

**Table 3:** Chemical composition of EOs from bee balm.

Peak	RT	RI^calc^	RI^lit^	Name	% of TIC					
					M.f. 1	M.f. 2	M.f. 3	M.f. 4	M.f. 5	M.r.
1	9.82	921	924	α-Thujene	2.85	1.09	1.00	2.13	2.10	0.86
2	10.02	930	932	α-Pinene	0.97	0.29	0.26	0.57	0.50	0.25
3	10.53	945	946	Camphene	0.27	0.09	0.12	0.10	0.09	0.07
4	11.30	968	969	Sabinene	0.18	0.06	0.09	0.14	0.17	0.09
5	11.45	972	974	β-Pinene	0.33	0.08	0.10	0.15	0.14	0.11
6	11.80	981	980	1-Octen-3-ol	1.88	1.00	1.19	1.96	2.20	1.43
7	11.96	990	988	Myrcene	2.13	0.88	0.92	1.59	2.08	0.60
8	12.44	1002	1002	α-Phellandrene	0.49	0.21	0.22	0.36	0.48	0.16
9	12.50	1006	1008	δ-3-Carene	0.18	0.10	0.09	0.15	0.19	0.08
10	12.85	1015	1014	α-Terpinene	3.80	1.91	1.46	2.39	3.92	1.64
11	13.23	1022	1020	p-Cymene	9.98	10.97	6.93	19.73	10.21	15.19
12	13.37	1024	1024	Limonene	4.50	0.65	0.35	0.77	1.03	0.47
13	13.40	1026	1025	β-Phellandrene	nd	nd	0.13	0.06	0.10	nd
14	14.28	1057	1054	γ-Terpinene	7.16	3.64	5.21	6.77	17.83	2.90
15	14.53	1064	1065	(Z)-Sabinene hydrate	0.28	0.52	0.36	0.69	0.47	0.63
16	14.98	1085	1086	Terpinolene	0.17	0.11	0.09	0.10	0.12	0.07
17	15.44	1096	1095	β-Linalool	0.42	0.06	0.10	0.05	0.14	0.11
18	15.64	1122	1119	(E)-Pinene hydrate	0.16	0.07	0.06	0.09	0.11	0.08
19	18.05	1171	1174	1-Terpinen-4-ol	0.61	0.09	0.19	0.43	0.53	0.79
20	18.99	1185	1186	α-Terpieol	0.13	0.11	0.08	0.30	0.10	0.12
21	19.30	1230	1232	Thymol methyl ether	0.15	0.33	nd	nd	nd	0.10
22	19.70	1240	1241	Carvacrol methyl ether	6.37	6.65	nd	nd	nd	4.54
23	20.61	1288	1287	Linalyl acetate	0.80	0.10	nd	nd	nd	0.13
24	21.67	1290	1289	Thymol	51.57	66.87	0.54	0.46	0.24	62.45
25	21.75	1300	1298	Carvacrol	1.12	0.91	78.08	55.42	50.79	1.97
26	23.42	1374	1374	α-Copaene	0.12	0.05	0.09	0.14	0.10	0.07
27	23.65	1390	1387	β-Bourbonene	0.08	0.09	nd	0.31	0.18	0.11
28	24.62	1419	1417	β-Caryophyllene	1.20	0.25	nd	0.10	1.90	0.32
29	25.95	1476	1478	γ-Muurolene	0.14	0.16	0.09	0.17	0.13	0.19
30	26.14	1486	1484	Germacrene D	0.73	0.44	0.88	1.98	2.28	0.69
31	26.48	1500	1498	α-Selinene	0.07	0.10	0.09	0.25	0.14	0.19
32	26.60	1505	1505	(E,E)-α-Farnesene	0.19	0.18	0.23	0.20	0.36	0.14
33	26.88	1512	1513	γ-Cadinene	0.06	0.09	0.15	0.12	0.08	0.70
34	26.99	1521	1522	δ-Cadinene	0.16	0.21	0.10	0.24	0.22	0.25
35	27.79	1533	1533	Thymohydroquinone	0.17	0.98	0.16	1.30	0.40	1.70
36	28.15	1580	1582	Caryophyllene oxide	0.11	0.07	0.05	0.10	0.09	0.14

RT—retention time; RI^calc^—relative index calculated from data; R^Ilit^—relative index from the literature data; TIC—total ion current.

**Table 4:** Chemical composition of EOs from mint.

Peak	RT	RI^calc^	RI^lit^	Name	% of TIC	
					M. l.	M. p.
1	9.82	921	924	α-Thujene	0.86	0.06
2	10.02	930	932	α-Pinene	0.88	0.07
3	11.30	968	969	Sabinene	3.57	0.25
4	11.45	972	974	β-Pinene	0.66	0.08
5	11.80	981	980	1-Octen-3-ol	nd	0.12
6	11.96	990	988	Myrcene	1.93	0.07
7	12.15	992	994	Octan-3-ol	0.20	nd
8	12.44	1002	1002	α-Phellandrene	0.09	nd
9	12.85	1015	1014	α-Terpinene	5.07	nd
10	13.02	1022	1020	p-Cymene	0.97	0.32
11	13.18	1024	1024	Limonene	0.76	3.03
12	13.22	1027	1026	Eucalyptol	1.81	0.50
13	13.40	1032	1032	β-cis-Ocimene	0.62	1.01
14	13.65	1043	1044	β-trans-Ocimene	nd	0.59
15	14.03	1057	1054	γ-Terpinene	7.82	0.10
16	14.54	1064	1065	(Z)-Sabinene hydrate	17.09	0.25
17	15.03	1085	1086	Terpinolene	1.80	1.36
18	15.18	1090	1090	p-Cymenene	nd	0.69
19	15.31	1095	1095	trans-β-Terpineol	0.91	0.98
20	16.24	1120	1119	trans-Mentha-2,8-dien-1-ol	0.56	0.12
21	16.69	1140	1130	cis-Myroxide	nd	1.15
22	16.76	1134	1133	cis-Mentha-2,8-dien-1-ol	0.22	0.11
23	17.29	1150	1148	Menthone	15.08	nd
24	17.53	1165	1167	Menthol	6.50	nd
25	18.05	1171	1174	1-Terpinen-4-ol	8.08	0.29
26	18.13	1177	1179	p-Cymen-8-ol	1.50	2.78
27	18.16	1180	1180	Isomentol	0.26	nd
28	18.35	1185	1186	α-Terpieol	0.32	1.53
29	18.41	1197	1195	cis-Piperitol	0.12	nd
30	18.76	1209	1207	trans-Piperitol	0.10	nd
31	18.94	1214	1213	trans-Pulegol	0.60	nd
32	19.18	1220	1220	cis-Sabinene hydrate acetate	0.09	0.16
33	19.31	1225	1225	Citronellol	nd	0.29
34	19.52	1229	1228	cis-p-Mentha-1(7),8-dien-2-ol	nd	0.14
35	19.60	1235	1233	Pulegone	2.18	0.16
36	20.18	1250	1249	Piperitone	0.11	8.68
37	20.53	1272	1271	neo-Menthyl acetate	0.20	0.12
38	20.95	1295	1294	Menthyl acetate	0.12	1.54
39	21.02	1300	1299	Terpinen-4-ol acetate	0.92	0.23
40	21.46	1307	1305	Isomenthyl acetate	0.57	0.10
41	22.41	1342	1340	Piperitenone	0.26	0.95
42	23.36	1374	1374	α-Copaene	0.09	0.36
43	23.54	1380	1379	Geranyl acetate	0.10	0.41
44	23.65	1385	1387	β-Bourbonene	0.20	0.35
45	23.75	1392	1390	β-Elemene	1.17	1.43
46	24.29	1410	1409	α-Gurjunene	nd	0.46
47	24.62	1419	1417	β-Caryophyllene	5.74	20.23
48	24.98	1430	1431	β-Gurjunene	nd	0.70
49	25.32	1440	1440	cis-β-Farnesene	0.27	0.19
50	25.54	1454	1452	α-Caryophyllene	0.52	1.45
51	26.06	1484	1483	Germacrene D	6.98	21.98
52	26.47	1521	1522	Bicyclogermacrene	0.89	0.27
53	26.61	1540	1540	α-Copaen-11-ol	nd	0.63
54	26.91	1512	1513	γ-Cadinene	0.13	0.55
55	27.01	1521	1522	δ-Cadinene	0.16	0.34
56	27.79	1550	1548	Elemol	0.18	0.26
57	27.93	1575	1577	Spathulenol	nd	1.70
58	28.11	1580	1582	Caryophyllene oxide	0.20	0.75
59	28.29	1590	1590	Globulol	nd	0.59
60	28.58	1604	1602	Ledol	nd	3.80
61	28.83	1620	1618	1,10-di-epi-Cubenol	nd	1.05
62	29.29	1627	1627	1-epi-Cubenol	nd	0.78
63	29.53	1639	1638	epi-α-Cadinol	nd	0.65
64	29.68	1645	1645	Cubenol	nd	1.23
65	29.97	1652	1651	α-Eudesmol	nd	1.07
66	30.02	1654	1653	α-Cadinol	nd	0.59
67	30.24	1696	1698	(2Z,6Z)-Farnesol	nd	0.67
68	32.88	1760	1759	Cyclocolorenone	nd	5.99
69	34.46	1845	1845	(2E,6E)-Farnesyl acetate	nd	1.14
70	34.56	1862	1860	(Z,Z)-Farnesyl acetone	nd	0.75
71	35.30	1913	1913	(5E,9E)-Farnesyl acetone	nd	1.21

RT—retention time; RI^calc^—relative index calculated from data; R^Ilit^—relative index from the literature data; TIC—total ion current.

**Table 5:** Chemical composition of EOs from marigold.

Peak	RT 2022/2021	RI^calc^ 2022/2021	RI^lit^ 2022/2021	Name	% of TIC			
					T.e.1-2022	T.e.2-2022	T.e-2022	T.p.-2021
1	9.90/10.02	930	932	α-Pinene	0.53	0.15	0.34	0.20
2	11.23/11.30	968	969	Sabinene	5.66	3.04	1.92	0.64
3	11.30/11.45	972	974	β-Pinene	0.31	0.15	0.23	0.10
4	11.77/11.96	990	988	Myrcene	0.22	0.27	1.03	0.12
5	12.31/12.44	1002	1002	α-Phellandrene	0.85	0.45	nd	0.22
6	13.00/13.01	1022	1020	p-Cymene	0.19	0.12	0.15	0.16
7	13.10/13.14	1024	1024	Limonene	10.29	4.35	9.28	6.68
8	13.31/13.65	1043	1044	β-trans-Ocimene	3.97	4.41	1.89	16.40
9	13.85/13.68	1047	1046	Dihydrotaghetone	7.85	8.80	8.20	1.71
10	14.02/14.28	1057	1054	γ-Terpinene	0.16	0.10	0.18	nd
11	14.51/14.48	1064	1065	(Z)-Sabinene hydrate	0.37	0.57	nd	nd
12	14.88/14.96	1085	1086	Terpinolene	2.95	1.54	27.61	13.40
13	15.04/15.12	1090	1090	p-Cymenene	1.14	0.72	1.75	0.76
14	15.32/15.42	1096	1095	β-Linalool	0.17	0.15	3.86	0.53
15	15.78/15.91	1130	1128	allo-Ocimene	0.20	0.14	2.03	0.32
16	16.71/16.68	1140	1139	trans-Tagetone	1.31	3.80	1.78	4.37
17	16.95/16.99	1149	1148	cis-Tagetone	6.15	10.10	7.53	3.78
18	17.80/18.05	1171	1174	1-Terpinen-4-ol	0.34	0.15	1.29	0.26
19	18.24/18.35	1185	1186	α-Terpieol	0.42	0.17	1.21	0.65
20	18.54/19.20	1222	1223	β-Citronellol	0.77	1.51	0.83	nd
21	19.34/19.24	1226	1226	cis-Ocimenone	11.77	7.02	0.36	13.26
22	19.28/19.28	1228	1227	Nerol	nd	0.10	0.44	nd
23	19.65/19.52	1236	1235	trans-Ocimenone	41.23	49.42	0.30	9.30
24	19.92/20.03	1250	1249	Piperitone	0.54	0.36	18.32	4.22
25	22.24/22.41	1342	1340	Piperitenone	0.13	nd	1.66	11.05
26	22.75/22.95	1365	1366	Piperitenone oxide	0.40	nd	0.64	0.20
27	24.43/24.55	1419	1417	β-Caryophyllene	0.11	0.37	1.91	5.39
28	25.98/26.11	1486	1484	Germacrene D	0.65	0.76	1.51	2.11
29	28.13/28.13	1580	1582	Caryophyllene oxide	0.14	0.26	0.42	0.45

RT—retention time; RI^calc^—relative index calculated from data; R^Ilit^—relative index from the literature data; TIC—total ion current.

## Data Availability

Data is contained within the article.
